# Supplementary material for: Are carbon emissions trading and green financial instruments synergistic? -Comprehensive quantitative research based on content analysis
Source: PLoS One. 2024 Mar 7;19(3):e0298601. doi: 10.1371/journal.pone.0298601 (PMC10919601; doi:10.1371/journal.pone.0298601)
Supplement: S1 Data — (ZIP) [file pone.0298601.s001.zip › Data/地级市Z_F工作报告.zip!/║■─╧/╒┼╝╥╜τ╩╨/└┤╫╘╣1⁄2╓┌║┼í╛┬φ┐╦╩2╛▌═°í┐_2016.docx]

各位代表：

　　现在，我代表市人民政府向大会作工作报告，请予审议，并请各位政协委员及其他列席人士提出意见。

　　一、“十二五”时期经济社会发展回顾

　　五年来，面对“三期叠加”的宏观环境，我们在市委的领导下，团结带领全市各族人民，重点实施“提质张家界打造升级版”战略，攻坚克难，奋发进取。

　　这五年，是我市综合实力显著增强的五年。我们始终坚持以经济建设为中心，千方百计抓项目，全力以赴稳增长，经济运行稳中向好，主要指标保持两位数增长，增速位居武陵山片区前列。到2015年，全市地区生产总值达到453亿元（预计，下同），年均增速超过10%。人均生产总值跻身全省前十位。规模工业增加值达到50亿元。固定资产投资达到300亿元。社会消费品零售总额达到177亿元，今年增速排全省第一。一般公共预算收入达到45.3亿元。“十二五”发展主要预期目标基本完成，全市综合实力迈上新台阶。

　　这五年，是我市城乡面貌显著改观的五年。我们始终坚持统筹城乡发展，大力实施“四基工程”，抓投入、补短板， 城乡建设日新月异。全市基础设施建设投资累计达230亿元。中心城区基础设施不断完善，城市旅游功能更加配套，建成区从28平方公里发展到近33平方公里。中心镇、特色镇承载能力不断增强，两个县城得到扩容提质。全市常住人口城镇化率从36.2%提高到46%。城镇污水处理率达到83.6%，中心城区垃圾无害化处理率达到100%。城乡环境明显改善，生态环境不断优化。

　　这五年，是我市旅游发展显著提升的五年。我们始终坚持旅游主导，突出“三星拱月，全域旅游”，张家界知名度和吸引力进一步增强。天门山晋级为国家5A景区，我市成为国家旅游综合改革试点城市。全市景点接待旅游人次从2405万增长到5050万。旅游总收入从125亿元增长到339亿元。2015年旅游主要指标增速创近十年新高，一年增加游客1165万人次、旅游总收入90亿元，稳居全国非省会城市前列。

　　这五年，是我市民生事业显著进步的五年。我们始终坚持民生优先，不断提高基本公共服务水平，人民群众满意度和获得感大幅提升。城乡居民人均可支配收入分别达到19600元、6970元，年均分别增长9.6%和10.7%。扶贫攻坚成果丰硕，22.8万农村贫困人口实现稳定脱贫。社会保障、教育、卫生、文化等社会事业全面发展。社会治安民意调查连续四年位居全省前四，成功蝉联“长安杯”。

　　取得这些成就，我们主要做了以下工作：

　　（一）坚定不移加快基础设施建设。我们抢抓机遇，以道路交通建设为重点，着力建设完善现代基础设施体系。张花高速公路建成通车，荷花机场新航站楼投入使用，黔张常高铁和张桑高速公路建设全面推进，长期制约我市发展的交通瓶颈取得历史性突破，桑植县从此将结束“无铁路、无高速、无国道”的历史。重大交通基础设施的建设，让张家界融入了大市场、大区域。五年来，我们着力加强中心城区建设，以“两带三区”建设为重点，扎实推进中心城区“双百工程”，改造提升城市道路49公里、小街小巷179条，配套完善地下管网217公里，城市绿化率达到39.7%、人均绿地8.8平方米。五年来，全市新改建干线公路239公里、农村公路1507公里，建制村基本通水泥或柏油路。新增小水电站11座，农村电网改造率达到95.6%。新建天然气管道397.3公里，城市规划区管网覆盖率达到80%。新建沼气池16万立方米，农村清洁能源入户率达到27.8%。所有乡镇和96%的行政村通宽带，移动手机信号实现全覆盖。新建城市防洪堤18公里，除险加固中小型水库177座，治理中小河流143.6公里。这些任务的完成，使全市城乡基础设施有了前所未有的改善。

　　（二）坚定不移推进产业转型升级。我们紧紧扭住产业发展这个重中之重，不断优化经济结构，推动产业转型升级。完成了天门山等一批景区的复合型改造，建成了华天、禾田居、阳光、碧桂园等一批高端休闲度假酒店，包装了《张家界24小时》、“户外天堂张家界”等一批特色项目，观光带动、多元发展的旅游产品体系初步形成。电商、物流、会展等新兴服务业蓬勃发展，限额以上服务企业增长了140%，服务业增加值年均增长11.2%。大力发展新型工业，制定实施工业 “135行动计划”，全市规模工业企业达到162家、增加值年均增长11.3%，工业税收增长了1.1倍。清洁能源、旅游商品、生物医药、新型建材等产业加快成长，富有区域特色的新型工业体系逐步形成。制定实施农业“523行动计划”，建设了十大现代农业生产基地，存池大鲵达到123万尾，商品蔬菜基地达到24万亩，茶叶、水果、畜禽规模养殖快速发展。新发展农民合作组织1020个，市级以上农业产业化龙头企业达到122家。全市产业发展水平明显提升。

　　（三）坚定不移深化改革开放。我们正确处理政府和市场的关系，充分发挥市场在资源配置中的决定性作用，向改革开放要红利，重点实施了10大类63项改革。完成了市、县政府机构改革，调整合并了30个乡镇，整合了食药监管力量，进一步理顺了张家界经开区管理体制。精简下放市级行政审批事项，减幅超过50%。旅游综合改革全面推进，武陵源核心景区实现区处合一，大旅游管理格局初步形成。国有企业法人治理结构不断完善，城市公交运营体制改革基本完成，西线旅游资源成功实施重组。市公共资源交易中心成立运行。商事制度改革不断深化，今年全市新发展市场主体同比增长54.74%。“大众创业、万众创新”深入推进，设立创业孵化基地8个，建立科技创新平台13个，实施国家、省科技项目228个，高新技术产业产值年均增长13%。对外开放合作不断扩大，海关挂牌开关，进出口商品本地检验检疫获批，口岸扩大开放，台胞实现落地签注，出入境航班和人次年均分别增长65.5%和81.8%。招商引资取得成效，成功引进知名企业30余家，累计实际利用外资3.3亿美元、内资187亿元。我市经济发展活力进一步增强，对外开放水平进一步提高。

　　（四）坚定不移优先改善民生。我们始终把改善民生作为政府工作的出发点和落脚点。市财政投入近300亿元用于民生建设，占一般公共预算支出的65%。累计建成保障性住房16625套，改造棚户区13007户、农村危房39108户，搬迁地质灾害隐患点居民792户，解决了48.27万人的饮水安全问题。城乡居民低保、农村五保供养标准、新农合支付限额等社会保障标准均实现逐年提升。各类教育协调发展，支持张家界航院、市高级技工学校完成整体搬迁，启动实施张家界一中新校区建设，建成省级义务教育合格学校170所，省级农村公办幼儿园55所。医疗卫生条件大幅改善，市级“三院”实施整合，市人民医院新院启动建设，县乡各类医疗机构改扩建基本完成。文体事业迅速发展，市博物馆、图书馆等重大公共文体设施开工兴建，区县公共图书馆和文化馆、乡镇文化站、农家书屋实现全覆盖。坚持创新社会治理，全面落实安全生产责任，亿元GDP安全事故死亡人数由0.33下降到0.07。全面推进社会治安综合治理，“平安张家界”建设成效明显。坚持最严生态环境保护，淘汰落后产能21.2万吨，全市森林覆盖率达到69.72%，生态环境已成为我市最好的资源、最大的优势。

　　(下转2版)　　 (上接1版)（五）坚定不移加强政府自身建设。我们在加快经济社会发展的同时，始终注重加强政府自身建设。深入开展群众路线教育实践活动和“三严三实”专题教育，加快政府职能转变，推进政企、政事、事企分开；推行阳光政务，简化办事程序，公开“权力清单”、“责任清单”，建立健全电子政务网络体系，提高了政务服务效率。深入推进依法行政，修订完善市人民政府工作规则、重大行政决策程序规则、合法性审查及听证制度，建立健全了城乡规划委员会、土地管理委员会等决策机制；坚持定期向人大、政协报告和通报工作，自觉接受人大及其常委会法律监督、工作监督和政协民主监督及社会各界监督，建议、提案办理满意率保持在99%以上。深入推进抓落实能力建设，坚持领导干部联系重点工程、重要工作制度，明晰岗位职责，规范权力运行，严格绩效考核，落实党风廉政建设各项规定，干部作风得到改进，政府执行力得到提升。

　　各位代表，奋斗蕴含艰辛，成绩来之不易。我市“十二五”所取得的成就，是省委、省政府和市委正确领导的结果，是市人大、市政协有效监督、鼎力支持的结果，是全市广大干部群众团结奋斗、共同努力的结果。在此，我代表市人民政府，向全市各族人民，向驻张部队、武警官兵和政法干警，向各民主党派、工商联、人民团体和各界人士，向所有关心支持张家界发展的朋友们，表示衷心的感谢！

　各位代表，五年来的丰富实践和积极探索，为我们积累了十分宝贵的经验，我们深深感受到，张家界的发展：

　　必须始终树立发展是第一要务的意识。“发展是解决一切问题的关键”。我们能总体上完成“十二五”规划的艰巨任务，让张家界从旅游城市迈向旅游大市，从贫困山区迈向小康大道，归根结底，就是靠发展。同样，实现“十三五”奋斗目标，推动张家界全面建成小康社会，基本建成国内外知名旅游胜地，还是离不开发展。因此，我们要坚定信心，保持定力，心无旁骛，致力发展。

　　必须始终把产业发展作为重中之重。产业是经济发展的基础，产业强则市强民富。我们坚持“产业兴市、产业强市”，把稳增长、调结构、惠民生的着力点放在产业发展上，不仅实现了经济健康持续发展，而且为社会进步提供了强有力的支撑。事实证明，抓产业就是抓关键、抓民生、抓脱贫。

　　必须始终坚持凝聚发展合力。干事凝聚人心，创业汇集合力。这几年，我们坚持党对经济工作的领导，充分调动各方面的积极性，把追求引导在“想干事”上，把智慧集中在“会干事”上，把价值体现在“干成事”上，形成了锐意进取、干事创业的浓厚氛围。正是全市上下齐心协力，认准了的事就一往无前，部署了的事就一抓到底，才解决了一批难事，办成了一批实事，干成了一批大事。

　　必须始终把群众共享发展成果放在首位。人民对美好生活的向往，就是我们的奋斗目标。我们坚持以民生为本，加大民生投入，顺应人民群众期盼，认真办好了一批事关群众切身利益的民生实事，让广大人民群众切实共享了发展成果。发展连着民生，民生连着民心。只有群众有了更多的获得感，我们的工作才有更大的成就感。

　　各位代表，成绩令人鼓舞，问题不容回避。我们应该清醒地看到，受宏观环境的影响，我市同样面临经济下行压力，“十二五”规划的规模工业增加值、社会消费品零售总额、全社会研发经费占GDP的比重等几个指标难以达到预期目标。今后几年，我市经济社会发展仍然面临不少困难和问题，主要是：经济总量不大、人均均量和发展质量不高的状况还没有根本改变；旅游带动作用还没有充分发挥，产业规模偏小，结构性矛盾突出，经济发展内生动力严重不足；城乡基础设施建设相对滞后，交通、能源、水利、信息等基础条件与“国内外知名旅游胜地”的要求还不相适应；公共服务设施基础薄弱，教育、卫生、文化、体育等公共服务供给不足、质量不高；人民群众生活总体水平仍然较低，部分群众生活还相当困难，脱贫攻坚和民生改善任重道远。这些问题，我们必须高度重视，切实加以解决。

　　二、“十三五”发展面临的形势和任务

　　“十三五”时期，是全面建成小康社会的决胜期，也是我市基本建成国内外知名旅游胜地的关键期。我们必须审时度势，奋勇前进。

　　国家宏观政策将给我市加快发展带来重大机遇。中央坚持稳中求进工作总基调，经济长期向好的基本面没有改变。国家实施“一带一路”、长江经济带等重大发展战略，我市具有良好的区位优势；经济进入新常态，国家注重供给侧结构性改革，我市面临良好机遇；国家推进精准扶贫，把革命老区、民族地区、集中连片贫困地区作为脱贫攻坚重点，我市打赢脱贫攻坚战有了良好条件；国家强调绿色发展理念，加大对重点生态功能区的转移支付，我市可持续发展有了良好保障。

　　全省重大部署将给我市加快发展创造有利条件。省委、省政府突出“一带一部”新定位，明确“三量齐升”新要求，坚定“五化同步”新路径，发展内涵和条件发生深刻变革，为我市加快发展指明了方向和路径。张吉怀高铁、桑龙高速等重大基础设施即将开工，将使我市加快成为武陵山片区交通枢纽，加快构建现代化立体大交通体系。我省“十三五”提出把旅游作为推动全省经济发展的强大引擎，明确“以张家界为重点，大力建设张吉怀精品生态文化旅游经济带”，必将促进我市旅游产业新一轮大发展。

　　良好发展势头将给我市加快发展增添必胜信心。进入“十三五”，我市将全面迈入高铁时代、智能化时代、创新创业时代、旅游经济时代，进入脱贫攻坚期、旅游黄金期、投资机遇期、产业转型期，全市基础设施日益改善，产业优势日益凸显，综合实力日益增强。特别是在市委的坚强领导下，全市上下和衷共济、团结一心，风清气正、心顺劲足，凝聚起了强大的发展正能量。只要适应新变化、把握新机遇，坚持变中求新、变中求进、变中突破，就必然开拓发展新境界，跨越发展新起点，迎来又一轮发展新高潮。

　　今后五年，政府工作的指导思想是：全面贯彻党的十八大和十八届三中、四中、五中全会精神，以邓小平理论、“三个代表”重要思想、科学发展观为指导，深入贯彻习近平总书记系列重要讲话精神，坚持“四个全面”战略布局，坚持“五个发展”新理念，按照全省“一带一部”新定位和“三量齐升”新要求，在市委的领导下，继续实施“一战略七行动”重点部署，力促旅游产业大市向旅游经济强市转变，确保全面建成小康社会、基本建成国内外知名旅游胜地目标圆满实现。

　　今后五年，全市经济社会发展的主要目标是：发展速度高于全省平均水平，居民收入增长高于经济增长，贫困人口收入增长高于居民平均收入增长；到2020年，地区生产总值突破700亿元，一般公共预算收入突破70亿元，旅游总收入突破700亿元。

　　经济平稳较快发展。经济结构优化升级取得重大进展，质量效益显著提升。地区生产总值年均增长9.5%，一、二、三产业增加值年均分别增长4%、12%、9%，规模工业增加值年均增长12%，到2020年三次产业结构比调整为9：27：64。一般公共预算收入年均增长10%。固定资产投资累计达2000亿元以上，年均增长18%以上。社会消费品零售总额年均增长12%，外贸进出口总额年均增长10%，综合实力再上新台阶。

　　旅游升级成效显现。加快实现旅游产品由观光型向多元复合型、旅游经济由粗放型向集约型、旅游产业由规模数量型向规模质量效益并重型转变。旅游主导地位更加突出，全省旅游龙头作用更好发挥，旅游产业国际化程度和品牌影响力不断提升。到2020年，力争全市旅游总收入、旅游接待人次、旅游过夜人数年均分别增长15%、10%、12%以上，基本建成国内外知名旅游胜地。

　　人民生活持续改善。城乡居民收入普遍较快增长，人民生活更加殷实，幸福指数较大提高。到2020年，城乡居民人均可支配收入分别突破31000元和11000元，年均分别增长10%和11%。年均新增城镇就业9000人以上，城镇登记失业率控制在4.5%以内，人民群众获得感进一步增强。

　　生态环境更加优良。资源利用效率明显提升，人居环境进一步改善。到2020年，森林覆盖率保持在70%，城市污水集中处理率达95%，生活垃圾无害化处理率保持100%，城市空气质量优良率达85%。国家森林城市创建达标，生态文明建设走在全省前列，成为国家生态文明建设示范区。

　　社会事业不断进步。覆盖城乡居民的公共服务体系和社会保障体系不断完善，城乡一体化发展格局基本形成。到2020年，城镇化率达56%；九年义务教育巩固率达到97%，基本普及高中阶段教育；城镇职工基本养老保险覆盖率达到100%，城镇职工和城镇居民基本医疗保险覆盖率达到100%，城乡居民养老保险覆盖率和新农合参合率均达到99%以上，总体实现城乡基本公共服务均等化。

　　开放水平明显提高。以国际视野规划城市，用国际标准建设城市，按国际惯例管理城市，“六城同创”和旅游城市国际化取得显著成效，市民素质和社会文明程度显著提升。更加有效拓展开放合作，在国际交流交往中提升发展水平，在区域合作共赢中抢占发展先机，对外交流合作继续走在全省前列。

今后五年，全市经济社会发展的基本原则是：

　　——坚持三量齐升、转型发展。适应发展新常态，抢抓战略机遇期，坚持速度与效益并重、发展与转型共进、当前与长远兼顾，以发展促进转型，以转型推动发展，促进经济总量、发展质量、人均均量同步提升。

　　——坚持改革驱动、创新发展。把改革创新作为引领发展的第一动力，充分发挥市场、政府和社会的积极作用，提高资源配置效率，勇于改革，不断创新，加快构建促进更大更快更好发展的产业体系和体制机制，使改革红利充分释放，发展活力充分迸发。

　　——坚持统筹协调、共享发展。坚持从实际出发，统筹城乡、区域协调发展。坚持以人为本，注重以民生为重点的社会建设，推进基本公共服务均等化，努力实现发展成果由人民共享。

　　——坚持突出特色、绿色发展。始终坚守生态环境这条生命线，大力推进生态建设，全面加强环境保护，促进资源节约和环境友好，走人与自然和谐共生的绿色发展之路，致力打造绿色、低碳、宜居、宜游的旅游城市。

　　今后五年，全市经济社会发展的重点是：

　　——加快推进精准脱贫。坚持区域发展与精准脱贫相结合，努力加快贫困地区和贫困群众脱贫致富奔小康步伐，确保到2020年，实现24.91万贫困人口整体脱贫、与全国全省同步全面建成小康社会。

　　——加快推进产业发展。坚持旅游主导地位，加快旅游转型提质升级，不断提高旅游经济综合竞争力。充分发挥旅游带动作用，促进旅游业与新型工业、现代农业、现代服务业和信息化深度融合发展，着力培育战略性新兴产业，加快形成以开放共享为特征的“旅游+”和“互联网+”产业模式，不断拓展具有旅游特色的产业融合发展新领域，推动产业扩量提质增效。

　　——加快推进新型城镇化。遵循旅游城镇发展规律，科学优化城镇空间资源，实施中心城区国际化战略，推进“智慧城市”建设，塑造城市风貌特色，增强城市人文魅力，加快建成和谐宜居、富有活力、特色鲜明的国际化山水型生态旅游城市。

　　——加快推进生态文明建设。科学布局生产空间、生活空间、生态空间，严格按照主体功能区定位推动发展，加快创建森林城市、生态城市，扎实推进生态环境保护，让良好生态环境成为人民生活质量的增长点，成为展现张家界良好形象的闪光点。

　　——加快推进社会建设。大力加强公共服务体系建设，着力解决好人民群众关心的教育、就业、收入、社保、医疗卫生、食品安全等问题。坚持多方参与、共同治理，统筹兼顾、动态协调的原则，完善社会治理格局，构建源头治理、动态管理和应急处置相结合的社会管理机制，形成社会治理和服务合力，促进社会治理现代化。积极开展交通模范城市、平安满意城市、文明城市、卫生城市等创建活动，力争社会治理、食药监管等工作继续走在先进行列，努力建设法治政府。

　　三、2016年的预期目标和工作重点

　　2016年是“十三五”规划实施的开局之年。全市上下要适应新常态，把握新形势，抢抓新机遇，凝心聚力、扎实奋斗，推动全市经济社会持续健康发展。

　　明年政府工作的总体要求是：以“四个全面”为总揽，深入贯彻落实中央、省委经济工作会议精神和市委重大部署，主动适应经济发展新常态，以“项目建设推进年”为重点，继续推进“一战略七行动”，稳增长、调结构、惠民生、防风险，努力实现“十三五”良好开局。

　　明年全市经济社会发展的主要预期目标是：地区生产总值超过500亿元、一般公共预算收入超过50亿元、旅游总收入超过400亿元，分别增长9.5%、10%、20%。景点接待旅游人次增长15%、过夜游客增长17%；固定资产投资增长20%；社会消费品零售总额增长12%；农业增加值增长4%，规模工业增加值增长9%；实际利用外资、内资分别增长10%；城乡居民人均可支配收入分别增长10%、11%。城镇登记失业率控制在4.5%以内，安全生产、人口自然增长率、节能减排完成省定目标。社会事业全面进步，民主法制全面加强，社会大局保持和谐稳定。

　　围绕上述要求和目标任务，全市将实施十大重点工程、十大产业项目、三十件民生实事，重点抓好以下七个方面的工作：

　　（一）突出项目攻坚，着力扩大有效投资。项目建设是经济工作的主要抓手，抓经济必须抓项目，加快发展必须大抓项目。要大力开展“项目建设推进年”活动，以项目带投入，以项目调结构，以项目增后劲。一是加快推进重大项目建设。全力抓好黔张常高铁、张桑高速公路、荷花大道、武陵山大道等十大重点工程建设，力争张吉怀高铁和桑龙高速公路等项目开工建设，进一步完善重大基础设施。全力抓好西线旅游开发、张家界茶叶开发、张家界国际家居广场建设等十大产业项目，进一步增强发展后劲。加快推进项目前期工作，力争宜张、张新高速公路和安张衡铁路等项目取得实质进展，力争宜张高铁列入国家“十三五”或铁路网中长期规划。二是着力强化要素保障。抓住中央适当扩大地方政府发债规模的机遇，用好专项建设基金、地方融资平台、债券市场等工具，更好发挥政府投资的引导作用。创新拓宽融资渠道，积极推行“PPP”、专项债券和产业基金等融资模式，努力扩大直接融资规模；积极防范金融风险，加强中小企业信用担保体系建设，规范融资平台，进一步扩大金融投放。完善土地利用总体规划，盘活存量土地，推进集约、节约用地，确保建设用地需要。依法依规强力推进征地拆迁，确保项目顺利开工。大力引进各类优秀人才，加强劳动技能培训，着力增强人力、智力支撑。三是切实提高项目建设水平。强化规划龙头地位，着力提升项目质量和品位。全面落实重大工程项目责任制度，加强项目调度、督导和考核，在全市上下形成大抓项目、抓大项目的浓厚氛围。致力改善施工环境，严格基本建设程序，不断优化政务服务，切实为项目建设保驾护航。

　　（二）突出转型升级，着力保持旅游经济持续增长。坚持旅游兴市，按照“提质核心、振兴东线、发展西线”的要求，努力构建全域旅游发展格局，致力打造国际知名旅游目的地。一是进一步加强旅游营销。坚持国际国内并重、政府市场结合、线上线下互动，不断增强营销工作的前瞻性和时代性。充分发挥企业、媒体等主体作用，加强城市之间旅游合作，进一步抓好“活动、媒体、事件、政策、客源地”等五大营销，巩固传统客源地市场，对接“一带一路”开拓新兴客源地市场，不断增强营销工作的针对性、专业性和实效性。二是大力推进旅游服务设施建设。把握旅游发展大势，加快核心景区提质改造，全力推进“厕所革命”，进一步提高景区接待能力和水平。适应游客多样性、个性化需求，着力抓好游客中心、停车场、旅游公路、旅游标识标牌等自驾游、自助游接待设施建设。主动应对项目建设对游客的影响，制定工作预案，科学组织分流，确保旅游的正常秩序。三是进一步强化旅游综合管理。深化“平安满意在张家界”专项活动，扎实推进全国旅游服务标准化试点城市建设。推进旅行社、旅游饭店、旅游客运等市场主体改革创新。加快旅游信息化建设，着力打造智慧旅游。加强旅游交通安全、食品安全管理。落实旅游执法联动、旅游投诉处理、旅游应急事故处理等工作机制，不断完善旅游应急指挥系统和旅游联合救援体系。严厉打击非法经营、扰乱市场秩序等不良行为，不断优化旅游市场环境。四是不断丰富旅游产品供给。大力拓展旅游线路，积极发展乡村旅游，促进旅游产品多样化发展。着力抓好特色街区、景区景点、休闲度假酒店等项目建设，积极培育一批有竞争实力的旅游企业。针对游客市场供求新趋势，打好“旅游+互联网”牌，大力发展在线旅游，推动旅游产品业态创新、发展模式变革、服务效能提高。打好“旅游+生态”牌，加快建设一批休闲度假、健康养老项目。打好“旅游+文化”牌，鼓励支持演艺业做优做强，深入挖掘本地历史文化，积极发展特色文化游。打好“旅游+商贸”牌，加快形成旅游与购物、物流、会展的联动发展格局。通过旅游产业的加快发展，增加更多就业岗位，让更多群众享受旅游发展成果。

　　（三）突出产业发展，着力增强综合经济实力。大力推进旅游业与新型工业、现代农业、现代服务业融合发展，不断优化产业结构，完善产业体系，提高质量和效益。一是做大新型工业。要充分发挥资源优势，立足本地实际，加快推进具有张家界特色的新型工业化。扶持中小企业发展，引导旅游装备企业发展，支持五倍子等生物医药资源开发利用，鼓励大鲵、蔬菜、茶叶等农产品精深加工，推进旅游商品产业园建设。加快三个工业园区发展，进一步完善园区体制机制，不断增强园区活力。进一步完善园区服务平台，加快标准厂房、园区道路、污水处理等项目建设，实现园区建设投资6亿元以上。培育壮大骨干企业，力争27家企业入规，新增一批过亿元企业。加快推进工业续建项目建设，尽快开工建设一批工业项目，力争新增入园企业20家、园区规模工业增加值增长12％。二是做优现代农业。紧紧依托旅游市场，发展规模种养和农产品加工，切实提升农产品附加值，不断提高农业比较效益。巩固提高水果、烟叶等优势产业，稳定发展茶叶、油茶、苗木花卉等特色农业。重点扶持大鲵和蔬菜产业发展，力争大鲵养殖规模达到160万尾、商品蔬菜基地达到26万亩。支持农业企业基地建设，加强农业产业园区建设，加快实施两个“百千万”工程。着力推进农产品批发市场和农贸市场建设，大力开展农超对接，不断拓宽农产品流通渠道。规范农村土地流转，促进土地适度集中，不断提高农业的规模效益。三是做活现代服务业。积极发展现代商贸物流业，开工建设南门口、仙人溪、桃花溪谷、大观园等特色街区，力争开通国际航空货运业务，大力发展电子商务。加快发展特色餐饮业，积极引进国内外知名餐饮品牌。培育发展会展业，支持会展服务企业专业化、品牌化发展。创新发展现代金融业，支持发展中小银行，积极发展村镇银行，加快推进慈利、桑植农村信用社股份制改革，支持、鼓励有条件的企业上市。不断拓展体育服务业，积极引导户外运动俱乐部健康发展。大力发展信息、科技等服务业，培育壮大健康养老、社区服务、中介服务等服务新业态。积极化解房地产库存，引导旅游地产、家庭旅馆健康发展。

　（四）突出城镇发展，着力加快新型城镇化步伐。坚持“山水相连，景城相依，产城互动”的理念，扎实推进新型城镇化。一是以“三化三改”为着力点，提升中心城区品位。突出中心城区重要道路、重要节点提质改造，全力实施城市亮化、绿化、美化工程，加快棚户区、道路设施、地下管网改造。突出沙堤和且住岗片区、市经开区、澧水两带的基础设施建设，加快澧水大桥、白马泉高架桥等“四路两桥”在建项目建设，全面启动武陵山大道、子午西路延伸段、天子山大道建设。巩固提升城市管理“八大行动”成果，深入推进城市精细管理，进一步提高游客和市民满意度。严格建筑渣土、城市垃圾管理，加强城区燃煤锅炉整治。深入推进国家森林城市创建，高质量完成公（游）园、城市道路、居民社区、机关庭院绿化建设任务。二是以扩容提质为重点，增强县城承载能力。大力支持县域城镇建设，统筹实施县城扩容提质。加大慈利蒋家坪新区、桑植老观潭新区建设力度，加快建设酉水风光产业带和老观潭文化产业园。三是以旅游小镇建设为突破口，构建特色城镇体系。加大资金投入，发挥资源禀赋，建设旅游小镇，打造旅游新亮点。突出地域特点，找准定位，错位发展，统筹推进重点镇建设，提高集镇综合承载能力，努力形成城镇相连、交通便捷、就业充分、生态宜居的城乡一体化发展格局。四是以美丽乡村创建为载体，加快建设社会主义新农村。按照生态宜居、生产高效、生活美好、人文和谐的要求，加快推进美丽乡村建设。深入挖掘历史文化、特色景观等资源，注重历史文化名村、传统村落保护，打造一批自然风貌独特、民族风情浓郁、产业发展突出的特色村寨。以推进“百城千镇万村”新农村工程为抓手，全面开展垃圾和污水治理。以清洁家园、清洁田园、清洁水源为重点，大力开展村庄人居环境综合整治。

　　（五）突出民生改善，着力提高群众生产生活水平。坚持把改善民生放在优先位置，以办好30件民生实事为抓手，多办为民的好事，办好利民的实事。一是强化社会保障。按照社会政策要托底的要求，织好社会保障网。继续扩大“五大保险”覆盖面，稳步提高各类社会保险待遇水平，确保社会化发放率达到100％。全面开展重特大疾病医疗救助工作，扩大救助对象范围，提高救助标准，切实保障困难群众基本生活。继续抓好敬老院改扩建和幸福院建设。推进阳光院建设，完成省定农村危房改造和各类保障性住房建设任务。大力促进社会就业，开展各类职业技能培训2万人次，力争新增城镇就业1万人、农村劳动力转移就业1.3万人。二是加快基本公共服务设施建设。基本公共服务是我市经济社会发展长期存在的短板，必须着力补齐、补优。加快教育事业发展，新建10所农村公益幼儿园，完成22所义务教育合格学校建设和20所薄弱学校改造任务，支持张家界一中新校区、张家界旅游学校和慈利、桑植职业中专建设。加快公共卫生事业发展，推进市人民医院新院、社区卫生服务中心、合格化示范乡镇卫生院等项目建设。理顺市、区县、乡、村卫生计生综合监督体制，全面完成卫生计生机构改革任务。加快文体事业发展，完善城乡文体基础设施，实现市博物馆开馆，大力开展送戏下乡、送电影下乡、全民健身等活动，不断丰富群众文体生活。三是加强生态环境保护。良好的生态环境是最公平的公共产品、最普惠的民生福祉。要切实加大森林资源和生物多样性保护力度，抓好森林防火、森林病虫害防治和地质灾害监测治理，启动森林防火重点项目三期和松材线虫防治二期工程建设，大力实施石漠化治理、退耕还林、生态公益林和天然林保护、矿山复绿等生态修复工程，加快实施核心景区移民搬迁工程。充分发挥国家主体功能区建设试点示范、全省首批低碳景区试点等平台作用，努力争取和用好用足用活相关政策，不断加快生态市建设进程。以大鲵保护区功能调整为契机，加强大鲵保护，促进大鲵开发。深入开展环境整治“6+1”行动，大力开展生态文明示范镇（村）和绿色学校、小区、酒店、家庭、机关等创建活动，实施100个村居家庭清洁行动。坚决淘汰落后产能、落后工艺，继续实施主要污染物排放总量前置审批、污染物排放许可证等制度，研究制定全市排污权有偿使用和交易制度。继续推进“两型”工业建设，加强对桑梓火电、南方水泥、万福药业等重点企业的环境监管，着力打造天更蓝、水更清、地更绿、空气更新鲜的宜居张家界。四是大力推进脱贫攻坚。以建成全面小康社会为目标，深入实施全市扶贫攻坚五年行动计划。继续以桑植县为主战场，重点做好“四基工程”与“四个切实”、“六个精准”、“五个一批”的结合文章，大力推进水、电、路、房、通信和环境保护“六到农家”，实行连片集中扶贫、精准脱贫，确保完成省定年度减贫任务。认真开展旅游扶贫试点，把扶贫开发与生态建设、新型城镇化、特色产业发展等结合起来，加快改变贫困地区面貌。

　　（六）突出社会治理，着力维护社会和谐稳定。全面加强和创新社会治理，不断提高社会治理能力和水平，努力实现人民安居乐业、社会安定有序。一是加大依法治理力度。大力推进法治张家界建设，深入开展“七五”普法，加强人民调解、行政调解、司法调解联动的衔接配合机制，做好社区矫正和安置帮教工作。加大矛盾纠纷排查和信访积案化解力度。　　　　　　 （下转4版）　　（紧接2版）完善法律援助制度，推进公共法律服务体系建设。二是加强社会治安防控体系建设。深入创建“平安张家界”，着力抓好社会治安综合治理基层基础工作。全面推行“网格化”管理，进一步完善立体化社会治安防控体系，加强网络舆情监测和应急处突能力建设，有效预防和严厉打击“黄、赌、毒”等违法犯罪活动，确保社会大局和谐稳定。三是强化公共安全管理。以提高群众安全感为己任，全面落实安全生产责任制。坚守安全底线，突出抓好旅游、道路交通、非煤矿山、特种设备、烟花爆竹、校车等重点领域和重点行业专项整治，从源头上预防和杜绝安全隐患。切实做好防汛抗旱和气象、地震等防灾减灾工作。加强学校、医院和公共娱乐场所等重点部位消防安全管理。建立最严格的食药监管制度，确保人民群众“舌尖上的安全”。

　　（七）突出改革开放，着力激活发展动力。坚持以改革创新促发展，大力破除影响发展、制约转型的体制机制弊端。一是推进关键环节和重点领域改革。全面完成合村改革。继续推动新一轮国有企业改革。深化商事制度、农村土地、价格体制、财税体制、金融体制、投融资体制、工业园区管理体制改革，加快推进不动产登记、公共资源管理体制、生态文明体制、行政执法体制等各项改革。着力抓好殡葬改革、公车制度改革。稳步推进公安改革、教育综合改革和供销合作社综合改革。二是形成创新创业机制。着力激活创新主体，积极培养引进创新型企业家、高技能人才、农村实用人才。着力夯实创新载体，重点围绕旅游业、特色现代农业、新材料、植物提取等领域，打造研发设计平台、基础条件平台、知识产权平台和创业孵化平台，构建一批低成本、便利化、全要素、开放式的众创空间，启动建设市科技馆。着力优化创新环境，依法加强知识产权保护，强化财政资金引导等公共服务保障。加强科普教育基地建设，不断提高市民科学素养和科技技能。三是提高对外开放合作水平。积极拓展口岸服务功能，力争获批旅游购物离境退税试点，提升口岸开放水平。积极创造条件，努力打造张家界外贸综合服务平台。充分发挥出口基地县和出口龙头企业作用，进一步扩大外贸规模。不断扩大对外合作交流，积极发展友城关系，不断拓宽合作领域。四是加大招商引资力度。创新招商方式、落实招商政策，形成大招商、招大商，真招商、招真商的浓厚氛围。围绕健康养老、生物医药等产业，大力引进上市公司、央企和国际知名品牌企业等战略投资者。继续坚持招商引资项目领导责任制和项目履约调度机制，力争全年签约重大项目30个，力促已签约项目落地开工建设。

　　四、切实加强政府自身建设，为“十三五”发展提供有力保障

　　实现“十三五”良好开局，完成“十三五”目标任务，我们必须加强政府自身建设。

　　第一，坚持解放思想，科学施政。适应新形势，加快张家界发展，必须进一步解放思想、转变观念，增强驾驭经济工作的能力。增强战略眼光。树立全球视野，强化战略思维，在思想观念、体制机制、方式方法上与时俱进，牢牢把握发展大势，切实增强工作主动性。主动认识、适应、引领新常态，全面把握变化中蕴含的新机遇，切实提高工作的敏锐性、预见性。增强创新意识。善于学习和总结新常态下推动经济社会发展的新理念、新方法、新经验，敢于突破陈规，用改革的办法破解难题、以创新的思路加快发展。增强大局观念。必须坚持党对经济工作的领导。坚持胸怀大局，像维护眼睛一样维护大局，善于团结协作。始终坚持全市“一盘棋”，相互配合、相互支持，主动出主意、想办法、作贡献，心往一处想、劲往一处使，凝聚起发展的强大合力。

　　第二，坚持严以用权，依法行政。牢固树立法治意识，把政府工作全面纳入法治轨道。自觉接受监督。自觉接受人大及其常委会的法律监督、工作监督和政协的民主监督，强化监察、审计等政府内部监督，重视司法监督，主动接受舆论、社会监督。切实加强同人大代表和政协委员的联系，认真办理好人大代表议案、建议和政协委员提案。规范权力运行。切实落实好“三公开、三清单”制度，确保权力在阳光下运行。严格规范公正文明执法，深化行政执法体制改革，完善行政执法程序，加强行政执法队伍建设，不断提高行政执法水平。大力加强政务服务窗口建设，深化行政审批制度改革，推进联合审批、联合验收，进一步优化审批服务流程，提高办事效率。强化民主决策。严格执行公众参与、专家论证、风险评估、合法性审查和集体讨论决定等法定程序，积极推行政府法律顾问制度，建立重大决策终身责任追究制及责任倒查机制，确保决策制度科学、程序正当、过程公开、责任明确。

　　第三，坚持务实担当，高效理政。牢固树立公仆意识，大兴求真务实之风，让真抓实干、克难攻坚、加快发展的观念化为自觉行动。要聚焦发展。坚持加快发展不动摇，始终牢记发展不足、结构不优是我市最大的市情，以强烈的使命担当、昂扬的精神状态、务实的工作举措，力促我市经济社会又好又快发展。要敢于担当。强化“实干就是能力、落实就是水平”的工作导向，倡导担当、干事、尽责的过硬作风。各级领导干部面对改革深层次矛盾，要迎难而上、勇挑重担；面对重大决策实施、重点工程推进、重点领域突破等任务，要站得出来、顶得上去，敢打攻坚战，敢啃硬骨头，做到真干事、敢干事、会干事。要强化执行。严格落实岗位对责、绩效对账的督查工作机制，提高督查深度和频率，对重大项目、重点工程、重要事项实行跟踪督办，一抓到底。深化行政效能建设，铁腕治庸治懒治散，切实解决不作为、慢作为等问题，切实提高执行力。要严格奖惩。加强行政效能监察和考核制度建设，严格考评，强化绩效评估考核结果运用，努力形成比工作、比实绩的生动局面。

　　第四，坚持转变作风，廉洁从政。牢固树立廉政意识，切实把纪律挺在前面，坚持作风建设常抓不懈，让为民务实清廉成为广大党员干部的自觉行动，凝聚抓发展、促发展的强大合力。要加强反腐倡廉。严格落实中央“八项规定”，自觉践行“三严三实”要求，坚决克服和整治“四风”，巩固和拓展党的群众路线教育实践活动成果。认真落实党风廉政建设主体责任和“一岗双责”，全面推进惩治和预防腐败体系建设，健全完善廉政风险责任落实、动态监控、预警处置和检查评估机制，从源头上预防和惩治腐败。要强化管理保廉。严格落实项目招投标、政府采购、国有土地出让等公共资源交易管理规定，把权力关进制度的笼子，用制度管权、按制度办事。加强审计监督、财政监督，加大对重点领域、关键环节的监察力度。强化经济责任、民生工程和重大投资项目审计，严肃查处各类违法违纪案件，坚决纠正损害群众利益的不正之风。要厉行节约促廉。全面落实厉行节约反对铺张浪费有关规定，强化财政资金监管，严控一般性支出，压缩“三公”经费规模，提高资金使用效率，把财政资金更多更好的用于推动发展、改善民生，以清正廉洁、勤俭节约、艰苦奋斗的本色凝聚力量、赢得民心。

　　各位代表，蓝图已经绘就，前景无限美好。让我们在市委的坚强领导下，咬定目标不放松，抢抓机遇不停步，只争朝夕，团结拼搏，为加快实现“旅游胜地梦”、“全面小康梦”而努力奋斗！
